# Toward a solution-focused addiction science

**DOI:** 10.3389/fpubh.2025.1701524

**Published:** 2025-11-25

**Authors:** William L. White, John F. Kelly

**Affiliations:** 1 Emeritus Senior Research Consultant, Chestnut Health Systems, Bloomington, IL, United States; 2 Recovery Research Institute, Massachusetts General Brigham, Academic Medical Centers, Boston, MA, United States; 3 Department of Psychiatry, Harvard Medical School, Boston, MA, United States

**Keywords:** addiction, recovery, remission, resilience, substance use disorder

## Abstract

During the past 50 years there has been immense progress in our understanding of the etiology, pharmacology, neurobiology, epidemiology, and typologies of alcohol and other drug use disorders that has given rise to numerous novel pharmacological and behavioral treatments and recovery support services. Consequently, much has been documented and published regarding the causes and effects of drug use, addiction, related pathologies, and myriad modes of treatment. While such progress is to be celebrated, significant challenges nevertheless remain. This paper describes a novel perspective that proposes to enhance the effectiveness of our international efforts in addressing endemic drug problems through rigorous investigation into the successful long-term solutions that exist within the lived experiences of individuals, families, and communities. In this regard, such solutions are proposed to exist on a continuum of change spanning five domains: *resistance*, *resilience*, *risk minimization*, *remission*, and *recovery,* with each viewed as an independent achievement or as stages within a larger process of personal change. Ultimately, it is argued that the frontiers of addiction research lie in extending our study of the problem to include an intense focus on the prevalence, pathways, processes, styles, and stages inherent within these five domains.

## Introduction

Recent decades have witnessed exponential growth in the volume and methodological rigor of addiction-related research studies (1). The depth of this new knowledge has occurred in areas of addiction etiology (e.g., genetics, epigenetics, trauma/stress), drug pharmacology, neurobiology, epidemiology, and typologies, that has resulted in greater elaboration and discovery of the exact biopsychosocial nature of the spectrum of substance involvement and impairment resulting in clearer delineation and diagnostic classification of these phenomena (2). The personal, familial, community, and cultural consequences of excessive drug use also have been meticulously studied and reported (3). Internationally and as a specialized field, we have developed and evaluated multimodality systems for the treatment of addiction, with a particular focus on risk factors related to addiction recurrence. Within the arenas of prevention, harm reduction and treatment, our focus has been on those individuals with the highest problem severity, complexity, and chronicity. In short, much has been documented and published with what we know about drug use, drug addiction, its related pathologies, and the many modes of addiction treatment.

The question to be addressed in this commentary is: How much is known scientifically about the long-term solutions to the problem of addiction—solutions that exist within the lived experience of individuals, families, and communities? For those at highest risk of severe and prolonged addiction careers, solutions might be conceived to exist on a continuum of change spanning five domains: *resistance*, *resilience*, *risk minimization*, *remission*, and *recovery*. Each domain may be viewed as an independent achievement or as stages within a larger process of personal change (4, 5, 47).

Definitions of these domains may be contested by those seeking to protect or extend the boundaries of each concept and the professional and economic interests contained in each (48). Rigorous evaluation of proposed definitions is warranted, but beyond our present intent. However, to assure both conceptual integrity and broad applicability, each proposed definition must eventually, and in practice, meet the criteria of precision, inclusiveness, exclusiveness, measurability, acceptability, and simplicity (6).

### Proposition and analysis

*Resistance* is the process through which those who know they may be at high risk of addiction consciously refuse or severely limit drug exposure as a preventative measure and as an assertion of personal or cultural sovereignty (7). Innumerable studies have identified such addiction risk factors as familial addiction history, early onset of drug use, atypical drug tolerance, developmental trauma, co-occurring medical/psychiatric illness, and drug-saturated physical and social environments (8). But from the standpoint of science, we know very little about the experiential psychodynamics of successful drug avoidance and refusal among those individuals at highest risk for substance use disorders. Such knowledge could enhance the design of targeted primary prevention programs.

Addiction is a disease of exposure; resisting individuals manage their personal risk by consciously refusing or limiting drug exposure and often doing so as an act of psychological defiance or social protest. This is a pattern sometimes seen in children traumatized by parental addiction (“I’ll never end up like my dad”) or among groups who have experienced the weaponization of drugs as a tool of cultural subjugation (9, 10). Examples of the latter can be found within two historically drug-besieged communities. Drug resistance within Native American tribal communities extends from early abstinence-based religious and cultural revitalization movements (e.g., Handsome Lake Movement, the Prophet Movements, Peyotism) to the more recent Red Road and Wellbriety movements (11). Drug resistance among African Americans can be found in calls for abstinence-based cultural protection and revitalization that span the writings of Frederick Douglass, Malcolm X, and other prominent leaders (12, 46). As a scientific community, we know very little about the life trajectory of high-risk individuals and groups who resist drug exposure. The high risk of intergenerational transmission of addiction and related problems linked to historical trauma is well-documented (13), but we know little about those experiencing such trauma who escape such transmission.

*Resilience*, a form of neuropsychological hardiness or immunity, is the process through which those at high risk for addiction avoid addiction in spite of significant drug exposure (14). Consider two siblings who share numerous addiction risk factors and similar experimentation with a broad menu of psychoactive drugs. One develops a severe, decades-long addiction and related medical sequelae while the other achieves a healthy life trajectory. Scientifically, we know a great deal about the addicted sibling, but very little about the non-addicted sibling. We need to understand the protective factors of the healthy brother that prevented the progression from drug use to drug addiction (15). Identification of such factors and designing intervention programs to enhance such factors at personal, family, and community levels could guide the future design of targeted prevention and early intervention programs (16, 17).

A solution-focused research agenda could provide answers to key questions (18). Is immunity a fixed state or something more dynamic that ebbs and flows in response to human ontogenesis and changing life circumstances or fluctuations in health status? Are there educational, medical, or psychological interventions for people with high vulnerability for addiction that can reduce or eliminate such vulnerability? If, for example, the essence of addiction vulnerability is at its core metabolic dysregulation and addiction recovery involves a restoration or creation of metabolic health, then studies of the neurobiology of long-term recovery could open new avenues of preventive treatments, including gene therapy (e.g., CRISPR-facilitated gene editing), immunotherapy (e.g., addiction vaccines), and medications that provide immunity to overdose or drug-related damage to body organs.

*Risk Minimization*, in the context of addiction, is the process through which addicted individuals alter the frequency, intensity, manner, and circumstances of their drug use to minimize drug-related harm to self, family, and community. Harm reduction as a public health philosophy and model of service intervention has increased internationally, spurred first by addiction-related HIV/AIDS transmission and more recently as a response to the acceleration in opioid overdose deaths (19, 20). While successful risk minimization has been documented via reductions in key indices of harm (21–23), the personal mechanisms of change undergirding such efforts remain unclear and whether such mechanisms are confined to certain expressions (phenotypes) or stages of addiction (i.e., individuals in pre-contemplative or contemplative stages of change who are not ready or wiling to stop drug use, may utilize and benefit from certain harm minimization services (24, 25). Identifying and isolating such factors, designing interventions based on those factors, and expanding such interventions at a community level remain important agendas for most communities. Similarly, at a community and cultural level, extreme energy has been expended on the causes behind the exponential rise in drug overdose deaths, but little actual data on factors related to the recent decline in such deaths (26). Isolating such factors, designing interventions based on those factors, and expanding such interventions at a community level remain important agendas for most communities. As a research community, we need to study drug users, and particularly addicted drug users, who are achieving successful harm minimization. For example, we have considerable knowledge of people who experience multiple opioid overdose hospitalizations but not about those who experience one such hospitalization and avoid future overdose incidents (27). To what can the latter success be attributed and can it be brought to scale at a community level within the drug-using population?

*Remission* is the process through which those individuals who meet diagnostic criteria for a substance use disorder cease or decelerate drug use and related consequences to the point that SUD diagnostic criteria are no longer met (2). This change process spans full remission (absence of all SUD symptoms) or partial remission (absence of some but not all SUD Symptoms) remission (2). While a critically important milestone in and of itself, individuals nevertheless can remain in a grey zone of somewhat static remission where there has been a subtraction of addiction pathology, but not much improvement in the kinds of quality of life, meaning and purpose, and social involvement and social contributions often associated with a more flourishing, “technicolored,” recovery (28). We know that remission durability is time dependent—that the rate of addiction recurrence is high during early stages of remission, and we have documented the propensity for prolonged addiction careers marked by recycling through diverse treatment approaches (29). We know a great deal about the long-term trajectory of substance use disorders and brief abstinence and moderation experiments, but far less about the trajectory of successful long-term remission from substance use disorders. What do we know about the neurobiology of remission initiation and remission maintenance? What happens physiologically and psychologically to those who achieve successful long-term stable SUD remission? What are the stages of change and active ingredients that mark such success? The addictions literature is filled with studies of short-term relapse and related risk factors, but in comparison only scant attention to the mechanisms of long-term relapse and stable remission, and even less to the prevalence and processes involved in the achievement of partial SUD remission.

*Recovery* is the process through which those who have experienced addiction achieve remission plus significant elevations in global health, social functioning, and community contribution (citizenship) (6, 30). Recovery can include a state of flourishing—dramatic elevations in quality of life, meaning and purpose, and social contribution (31–35). Recovery is a recently emerging paradigm within efforts to forge more solution-focused systems of care for SUDs (36, 37). A recovery focused research agenda is just emerging as the scientific measurement of addiction recovery is in its infancy (4, 38). Most studies of recovery to date use measures of remission (pathology subtraction) and only recently has attention been given to what is added – the broader recovery-derived achievements in quality of life, social functioning, and community contribution (39, 48). Arriving at measurable operational definitions of recovery as distinct from remission [e.g., (40)], involving the different elements’ dimensional thresholds and durations within the recovery construct domain, is difficult but once achieved could lead to estimations of the prevalence of recovery (distinguished from remission) across demographic and clinical populations and how these rates are changing over time at local and national levels [e.g., (41, 42)]. Other questions to be answered include: What are the pathways of recovery initiation and the primary and secondary catalysts of change within these pathways? What varieties exist in the methods of successful long-term recovery maintenance? Are there specific recovery phenotypes as well stages of recovery and stage-specific challenges and opportunities across and within types? What are the stages and processes of family recovery? Does parental/family recovery reduce SUD risk for the children of formerly addicted parents?

Extracting the most potent and actionable ingredients from the collective experience of recovering individuals and their families could lead to bold innovations in the prevention and treatment of addiction as well as to new approaches to long-term recovery management (18). For example, is the prevalence of flourishing within addiction recovery greater within programs that integrate medication support within a vibrant, peer-based culture of recovery compared to approaches that only provide medication or provide only limited psychosocial support? As with remission, few references can be found to partial recovery in the addiction literature, and most of these refer to partial restoration of damaged body functions and not to SUD recovery.

It is important that solutions be explored across the spectrum of problem severity and complexity. For example, the prevalence of opioid overdose deaths is often cited as consequence of opioid addiction in spite of evidence that many people experiencing such deaths are opioid experimenters/users who would not meet diagnostic criteria for an opioid use disorder. We know very little about this population of drug casualties. How do drug experimenters/users avoid harm and progression to drug addiction?

Recent studies reveal that some chronic smokers avoid smoking-related illnesses (i.e., cancer) potentially due to genetically influenced protective mechanisms (43). There may be similar genetic protective factors that explain how some individuals can exhibit prolonged heavy use of other psychoactive drugs without developing a substance use disorder or drug-related medical complications. Isolating such factors through scientific inquiry might afford an opportunity for new interventions at personal and community levels to prevent or abort progression from drug use to destructive patterns of addiction. The research of Granfield and Cloud (44) underscores the importance of “recovery capital” in the resolution of severe substance use disorders, but such internal and external assets likely also apply to the successful resolution of subclinical substance-related problems (35). We need a deeper scientific exploration of the role of such assets within the prevention and resolution of drug-related problems across the severity spectrum (45).

## Discussion

As described extensively in the history of addiction treatment and related problems in America (1), the evolution of addiction science could be conceived to be marked by three broad stages. The first stage followed a pathology paradigm in which addiction was isolated as a medical and social problem worthy of scientific study. The focus was on the problem—its definition, classification, essential nature, patterns and prevalence, and its causes. The assumption was that if we expanded our knowledge of the sources of addiction—its root cause(s)—that solutions would emerge from that understanding. The second stage could be considered an intervention paradigm through which intervention programs were designed and evaluated across the continua of primary prevention, harm reduction, early intervention, treatment, and recovery support services. The assumption here was that identifying the most effective interventions would allow replication of the most effective interventions in local communities and across the world. Such identification and replication have occurred, but with increased understanding of their limitations. The third and emerging paradigm is study of the successful solutions at individual, family, community, and cultural levels that exist across the spectrum of drug problem severity and across diverse cultural contexts (6, 18). The assumption here is that studies of successful recovery trajectories and successful programs could reveal elements that could be broadly replicated in other service settings and population-based prevention efforts (41).

Review of existing literature in the domains above, highlights the vital and important progress that has been made in the past 100 years in addressing endemic addiction problems. It also uncovers and identifies substantive knowledge gaps and, consequently, many new opportunities. It is time as a research community that we developed an agenda that can broaden our scientific knowledge of all these levels of positive change. Each of the five domains of change described above we assert may exist on spectrums of depth and durability. The qualitative and quantitative dimensions of these changes warrant mapping and measurement as a future research agenda. We have measured what addiction has taken, but we have not measured the magnitude and types of positive changes that can occur across these five domains at personal, family and community levels (31, 33). We argue that expanding the frontiers of addiction research involves extending our study of the problem to include an intense focus on these solutions as noted above: the prevalence, pathways, processes, styles, and stages of resistance, resilience, risk minimization, remission, and long-term recovery. Scientists of our generation have entered this new solution-focused research frontier. It is our hope that the next generation of investigators will continue this exploration far beyond what we have envisioned to uncover, discover, and disseminate new knowledge of the multiple successful pathways followed by tens of millions into stable addiction recovery.

## Data Availability

The original contributions presented in the study are included in the article/supplementary material, further inquiries can be directed to the corresponding author.

## Author’s note

Adapted from William White Presentation at the 18th Addiction Science Conference, September 2–5, 2025, Tehran, Iran.

## References

[1] White WL. Slaying the dragon: The history of addiction treatment in America. Second ed. Bloomington, ILL: Chestnut Health Systems (2014).

[2] American Psychiatric Association. Diagnostic and statistical manual of mental disorders. 5th ed APA (2022).

[3] U.S. Department of Health and Human Services (HHS) Office of the Surgeon General. Facing addiction in America: The surgeon general's report on alcohol, drugs, and health. Washington, DC: HHS (2016). 28252892

[4] Kelly JF Bergman B Hoeppner B White WL. Prevalence, pathways, and predictors of recovery from drug and alcohol problems in the United States population: implications for practice, research, and policy. Drug Alcohol Depend. (2017) 181:162–9. 29055821 10.1016/j.drugalcdep.2017.09.028 PMC6076174

[5] Hser YI Evans E Grella C Ling W Anglin D. Long-term course of opioid addiction. Harv Rev Psychiatry. (2015) 23:76–89. doi: 10.1097/hrp.0000000000000052, PMID: 25747921

[6] White WL. Addiction recovery: its definition and conceptual boundaries. J Subst Abus Treat. (2007) 33:229–41. doi: 10.1016/j.jsat.2007.04.015, PMID: 17889295

[7] McCrady BS Flanagan JC. The role of the family in alcohol use disorder recovery for adults. Alcohol Res. (2021) 41:06. doi: 10.35946/arcr.v41.1.06, PMID: 33981521 PMC8104924

[8] Community Preventive Services Task Force. Excessive alcohol consumption. The community guide. Washington, D.C. (2023).

[9] Jilek WG. Native renaissance: the survival and revival of indigenous therapeutic ceremonials among north American Indians. Transcult Psychiatr Res Rev. (1978) 15:117–47. doi: 10.1177/136346157801500201

[10] Westermeyer J Neider J. Predicting treatment outcome after ten years among American Indian alcoholics. Alcohol Clin Exp Res. (1984) 8:179184. doi: 10.1111/j.1530-0277.1984.tb05833.x, PMID: 6375429

[11] Coyhis D White W. Alcohol problems in native America: The untold story of resistance and recovery. Colorado Springs, CO: White Bison, Inc. (2006).

[12] White W Sanders M Sanders T. Addiction in the African American community: the recovery legacies of Frederick Douglass and Malcolm X. Counselor. (2006) 7:53–8.

[13] Brave Heart MY. The historical trauma response among natives and its relationship with substance abuse: a Lakota illustration. J Psychoactive Drugs. (2003) 35:7–13. 12733753 10.1080/02791072.2003.10399988

[14] Rudzinski K McDonough P Gartner R StrikeIs C. Is there room for resilience? A scoping review and critique of substance use literature and its utilization of the concept of resilience. Subst Abuse Treat Prev Policy. (2017) 12:41 28915841 10.1186/s13011-017-0125-2 PMC5603070

[15] Rogers A Leslie F. Addiction neurobiologists should study resilience. Addict Neurosci. (2024) 11:100152. doi: 10.1016/j.addicn.2024.100152

[16] Calpe-López C Martínez-Caballero MA García-Pardo MP Aguilar MA. Resilience to the effects of social stress on vulnerability to developing drug addiction. World J Psychiatry. (2022) 12:24–58. doi: 10.5498/wjp.v12.i1.24, PMID: 35111578 PMC8783163

[17] Schasre R. Cessation patterns among neophyte heroin users^1^. Int J Addict. (1966) 1:23–32. doi: 10.3109/10826086609026749

[18] Kelly J. Tens of millions successfully in long-term recovery—let us find out how they did it. Addiction. (2017) 112:762–3. doi: 10.1111/add.13696, PMID: 28054421

[19] O’Hare P. Merseyside, the first harm reduction conferences, and the early history of harm reduction. Int J Drug Policy. (2007) 18:141–4. doi: 10.1016/j.drugpo.2007.01.003, PMID: 17689357

[20] Pridgen BE Bontemps AP Lloyd AR . U.S. substance use harm reduction efforts: a review of the current state of policy, policy barriers, and recommendations. Harm Reduct J. (2025) 22:101. doi: 10.1186/s12954-025-01238-4, PMID: 40484964 PMC12147315

[21] Logan DE Marlatt GA. Harm reduction therapy: a practice-friendly review of research. J Clin Psychol. (2010) 66:201–14. doi: 10.1002/jclp.20669, PMID: 20049923 PMC3928290

[22] Ritter A Cameron J. A review of the efficacy and effectiveness of harm reduction strategies for alcohol, tobacco and illicit drugs. Drug Alcohol Rev. (2006) 25:611–24. doi: 10.1080/09595230600944529, PMID: 17132577

[23] Zolopa C Clifasefi SL Dobischok S Gala N Fraser-Purdy H Phillips MK . A scoping review of harm reduction practices and possibilities among indigenous populations in Australia, Canada, and the United States. Drug Alcohol Depend. (2025) 269:112597. doi: 10.1016/j.drugalcdep.2025.112597, PMID: 39970577

[24] Dowell D Noonan RK Houry D. Underlying factors in drug overdose deaths. JAMA. (2017) 318:2295–6. doi: 10.1001/jama.2017.15971, PMID: 29049472 PMC6007807

[25] Rudd RA Seth P David F Scholl L. Increases in drug and opioid-involved overdose deaths — United States, 2010–2015. MMWR Morb Mortal Wkly Rep. (2016) 65:1445–52. doi: 10.15585/mmwr.mm655051e1, PMID: 28033313

[26] Stringfellow EJ Lim TY Humphreys K DiGennaro C Stafford C Beaulieu E . Reducing opioid use disorder and overdose deaths in the United States: a dynamic modeling analysis. Sci Adv. (2022) 8:eabm8147. doi: 10.1126/sciadv.abm8147, PMID: 35749492 PMC9232111

[27] Scott CK Dennis ML Grella CE Nicholson L Sumpter J Kurz R . Findings from the recovery initiation and management after overdose (RIMO) pilot study experiment. J Subst Abus Treat. (2020) 108:65–74. doi: 10.1016/j.jsat.2019.08.004, PMID: 31493942 PMC6893133

[28] McGaffin BJ Deane FP Kelly PJ Ciarrochi J. Flourishing, languishing and moderate mental health: prevalence and change in mental health during recovery from drug and alcohol problems. Addict Res Theory. (2015) 23:351–60. doi: 10.3109/16066359.2015.1019346

[29] White WL. Recovery/remission from substance use disorders: An analysis of reported outcomes in 415 scientific studies, 1868–2011 Great Lakes Addiction Technology Transfer Center. Chicago, IL. (2012).

[30] Betty Ford Institute Consensus Panel. What is recovery? A working definition from the Betty ford institute. J Subst Abus Treat. (2007) 33:221–8. 10.1016/j.jsat.2007.06.001 17889294

[31] Gutierez D Goshorn JR Dorais S. An exploration of thriving over time in recovery. J Subst Abus Treat. (2022) 132:108612 10.1016/j.jsat.2021.108612 34489158

[32] Hart KE Singh T. An existential model of flourishing subsequent to treatment for addiction: the importance of living a meaningful and spiritual life. Illn Crisis Loss. (2009) 17:125–47. doi: 10.2190/IL.17.2.d

[33] Makin P Allen R Carson J Bush S Merrifield B. Light at the end of the bottle: flourishing in people recovering from alcohol problems. J Subst Use. (2021) 27:107–14. doi: 10.1080/14659891.2021.1905092

[34] Parker P Banbury S Chandler C. The utility of measuring flourishing in substance and alcohol use disorders research: a systematic review. Europ J Appl Posit Psychol. (2018) 2:1–13.

[35] White W Kurtz E. The varieties of recovery experience. Int. J. Self Help Self Care. (2006) 3:21–61.

[36] Best DW Lubman DI. The recovery paradigm: a model of hope and change for alcohol and drug addiction. Aust Fam Physician. (2012) 41:593–7. PMID: 23145400

[37] White WL. Recovery: its history and renaissance as an organizing construct. Alcoholism Treat Q. (2005) 23:3–15.

[38] White W. L. The future of recovery research. 17th international congress on addiction science, Razi convention hall, Tehran, Iran. (2025) Available online at: https://deriu82xba14l.cloudfront.net/file/2472/2025%20Future%20of%20Recovery%20Research%20Iran.pdf (Accessed February 13, 2025).

[39] Eddie D White W Vilsaint C Bergman BG Kelly J. Reasons to be cheerful: personal, civic, and economic achievements after resolving an alcohol or drug problem in the United States population. Psychol Addict Behav. (2021) 35:402–14. doi: 10.1037/adb0000689, PMID: 33764087 PMC8184567

[40] Hagman BT Falk D Litten R Koob GF. Defining recovery from alcohol use disorder: development of an NIAAA research definition. Am J Psychiatry. (2022) 179:807–13. doi: 10.1176/appi.ajp.21090963, PMID: 35410494

[41] Kelly JF Belisario K MacKillop J. Prevalence, predictors, correlates, and dynamic changes in the NIAAA-defined ‘recovery’ definition. Alcohol Clin Exp Res. (2025) 1–13. doi: 10.1111/acer.70172 PMC12638283 40990872

[42] Kelly JF Volkow ND Koh HK. The changing approach to addiction - from incarceration to treatment and recovery support. N Engl J Med. (2025) 392:833–6. doi: 10.1056/NEJMp2414224, PMID: 39991938

[43] Oh AL Mularski RA Barjaktarevic I Barr RG Bowler RP Comellas AP . Defining resilience to smoking-related lung disease: a modified Delphi approach from SPIROMICS. Ann Am Thorac Soc. (2021) 18:1822–31. doi: 10.1513/AnnalsATS.202006-757OC, PMID: 33631079 PMC8641833

[44] Granfield R Cloud W. Institutional growth of recovery capital In: Best D Hennessy E, editors. The handbook of recovery capital: Understanding the science and practice. Bristol, UK: Bristol University Press (2025). 10–33.

[45] Tolomeo S Baldacchino A Volkow ND Steele JD. Protracted abstinence in males with an opioid use disorder: partial recovery of nucleus accumbens function. Transl Psychiatry. (2022) 12:81. doi: 10.1038/s41398-022-01813-4, PMID: 35217657 PMC8881207

[46] White W Sanders M. Addiction and recovery among African Americans before 1900. Counselor. (2002) 3:64–6.

[47] Flaherty M. Psychological aspects of substance use disorders, treatment, and recovery. In eds. A. Douaihy and D. C. Daley. Substance use disorders. Oxford University Press. (2014) 27–61.

[48] Kelly JF. Hoeppner B. A biaxial formulation of the recovery construct. Addiction Research & Theory. (2014) 23:5–9. doi: 10.3109/16066359.2014.930132 PMID: 23145400

